# Population-based evaluation of a suggested anatomic and clinical classification of congenital heart defects based on the International Paediatric and Congenital Cardiac Code

**DOI:** 10.1186/1750-1172-6-64

**Published:** 2011-10-03

**Authors:** Lucile Houyel, Babak Khoshnood, Robert H Anderson, Nathalie Lelong, Anne-Claire Thieulin, François Goffinet, Damien Bonnet

**Affiliations:** 1 Hôpital Marie-Lannelongue, CMR-M3C, Université Paris-Sud, 133 avenue de la Résistance, 92350 Le Plessis-Robinson, France; 2 Inserm, UMR S953, Recherches épidémiologiques sur la santé périnatale et la santé des femmes et des enfants, UPMC, Université Paris-6, Paris, France; 3 Institute of Human Genetics, Newcastle University, Newcastle upon Tyne, UK; 4 CMR-M3C, Hôpital Necker-Enfants Malades, Paris, France; 5 Université Paris-Descartes, Paris, France

## Abstract

**Background:**

Classification of the overall spectrum of congenital heart defects (CHD) has always been challenging, in part because of the diversity of the cardiac phenotypes, but also because of the oft-complex associations. The purpose of our study was to establish a comprehensive and easy-to-use classification of CHD for clinical and epidemiological studies based on the long list of the International Paediatric and Congenital Cardiac Code (IPCCC).

**Methods:**

We coded each individual malformation using six-digit codes from the long list of IPCCC. We then regrouped all lesions into 10 categories and 23 subcategories according to a multi-dimensional approach encompassing anatomic, diagnostic and therapeutic criteria. This anatomic and clinical classification of congenital heart disease (ACC-CHD) was then applied to data acquired from a population-based cohort of patients with CHD in France, made up of 2867 cases (82% live births, 1.8% stillbirths and 16.2% pregnancy terminations).

**Results:**

The majority of cases (79.5%) could be identified with a single IPCCC code. The category "Heterotaxy, including isomerism and mirror-imagery" was the only one that typically required more than one code for identification of cases. The two largest categories were "ventricular septal defects" (52%) and "anomalies of the outflow tracts and arterial valves" (20% of cases).

**Conclusion:**

Our proposed classification is not new, but rather a regrouping of the known spectrum of CHD into a manageable number of categories based on anatomic and clinical criteria. The classification is designed to use the code numbers of the long list of IPCCC but can accommodate ICD-10 codes. Its exhaustiveness, simplicity, and anatomic basis make it useful for clinical and epidemiologic studies, including those aimed at assessment of risk factors and outcomes.

## Background

Because of the diversity of the cardiac phenotypes, classification of the overall spectrum of congenital cardiac defects has always been challenging, with the challenge exacerbated by the oft-complex association of intracardiac and extracardiac defects. The more complex the pathology, nonetheless, the more important is the need for specialists to speak a common language, and to unify the diagnostic process.

Two systems of classifications for coding and establishing medical and administrative databases for congenital heart defects (CHD) are currently used globally: the 10^th ^revised version of the *International Classification of Diseases *(ICD-10) [1], and the International Paediatric and Congenital Cardiac Code (IPCCC), the latter designed in particular for evaluating the results of congenital cardiac surgery [2].

ICD-10 was created by the World Health Organization to "permit the systematic analysis, the interpretation and the comparison of the mortality and morbidity data harvested in different countries or regions at different periods of time" [1]. It is based on the frequency of the various groups of diseases. The group of congenital anomalies, and particularly CHD, is poorly detailed, and includes many doublets and inaccuracies. Hence, this classification, despite its wide use by non-specialists, is increasingly considered inadequate by paediatric cardiologists and cardiac surgeons for describing the manifold congenital cardiac malformations [3-5].

During the 1990's, the Society of Thoracic Surgeons (STS) and the European Association for Cardio-thoracic Surgery (EACTS), and the European Association for Paediatric Cardiology (AEPC), independently developed systems of nomenclature for evaluating the diagnosis and outcomes of patients with congenitally malformed hearts. The resulting International Congenital Heart Surgery Nomenclature and Database was published in 2000 [6], simultaneously with the publication of the resulting European Paediatric Cardiac Code [7]. The International Working Group for Mapping and Coding of Nomenclatures for Paediatric and Congenital Heart Disease, also known as the Nomenclature Working Group, was then established to unify the 2 systems. This was achieved in 2005 by cross-mapping the two previous systems of nomenclature and creating the IPCCC http://www.ipccc.net[8]. The IPCCC has now been used by three systems for analyzing the surgical outcomes of patients with CHD according to case complexity, the Risk Adjustment in Congenital Heart Surgery-1, (RACHS-1) [9], the Aristotle score [10], and the STS-EACTS Score [11]. In the IPCCC, each individual lesion is coded with a six-digit numerical code. The long list details all known malformations, along with their multiple anatomic and clinical variants, with thousands of items arranged in seven main categories according to the first two numbers of the six-digit code. Several complementary short lists are available that are designed to be used for audit and research purposes; these short lists contain hundreds of terms [2].

The advantages of the IPCCC, particularly the long list, include its precision and exhaustiveness, since it excludes all doublets and ambiguities. At the same time, its complexity, with more than 10,000 codes, renders difficult the search for the code of an individual lesion.

Our aim in preparing our anatomic and clinical classification of congenital heart defects, or ACC-CHD, was to design a comprehensive classification that is easy to use, but which is based on the long list of IPCCC. We have achieved this by regrouping the lesions in a fashion that, at least to us, makes both anatomical and clinical sense. The proposed classification can be useful for conducting epidemiological and clinical studies of risk factors and outcomes, as it aims to incorporate different but complementary approaches based on pathology and clinical practice, as well as echocardiography and criteria used for surgical management.

## Methods

### Data source

We applied our classification to data obtained from a population-based epidemiological study of patients with congenitally malformed hearts (the EPICARD study) in France. EPICARD is an ongoing prospective cohort follow-up study of all children with a CHD born to women in the conurbation of Greater Paris between 2005 and 2008. Over that period, the total number of births in the conurbation was approximately 300,000. We included all cases of CHD, not only live births, but also stillbirths and terminations of pregnancy. The principal objectives of EPICARD are to use population-based data from a large cohort of patients with CHD to first, estimate total and live birth prevalence, pre- and postnatal diagnosis of CHD; second, assess medical and surgical management of children with CHD; third, evaluate neonatal mortality and morbidity and neuro-developmental outcomes of children with CHD; and fourth, identify the factors associated with their health outcomes, especially the role of events during the neonatal period and of the initial medical and surgical management.

The total number of births in the Greater Paris conurbation over the study period was 317,538. All cases, including live births, pregnancy terminations, and foetal deaths, therefore, if diagnosed in the prenatal period or up to one year of age in the birth cohorts between May 1^st ^2005 and April 31^st ^2008, were eligible for inclusion. The total number of cases included in the study was 2867, including 2349 newborns (82%), 465 pregnancy terminations (16.2%) and 53 foetal deaths (1.8%). Diagnoses were confirmed in specialized paediatric cardiology departments, and for the majority of pregnancy terminations and foetal deaths by pathological examination. For those instances in which a pathologic study could not be achieved, the diagnoses were confirmed by consensus by a paediatric cardiologist and a specialist in echocardiography, based on the results of prenatal echocardiographic examination.

### Design of the classification

We regrouped the malformations into 10 main categories and 23 subcategories, according to a multi-dimensional approach encompassing anatomy, echocardiography, and criteria for therapeutic management (see Additional File 1: Anatomic and clinical classification of congenital heart defects (ACC-CHD) with the corresponding IPCCC and ICD-10 codes). We deliberately chose not to take into account any presumed developmental mechanisms, because of the current lack of sufficient evidence permitting the linkage of these mechanisms with the observed phenotypes.

a) The ten main categories

The ten main categories were listed according to flow of blood.

1. Heterotaxy, including isomerism and mirror-imagery

We chose to regroup in this category all the anatomic-echocardiographic sequences known under the terms right or left isomerism, or polysplenia and asplenia syndromes, or visceral heterotaxy. The suggested definition for heterotaxy of the Nomenclature Working Group is: "an abnormality where the internal thoraco-abdominal organs demonstrate abnormal arrangement across the left-right axis of the body... heterotaxy does not include patients with either the expected usual or normal arrangement of the internal organs along the left-right axis, also known as "situs solitus", nor patients with complete mirror-imaged arrangement of the internal organs along the left-right axis also known as "situs inversus [12]. We chose, however, to include mirror-imagery, or situs inversus, in this group. This inclusion is because, first, the mirror-imaged arrangement may be considered a form of "heterotaxy" in that it is a departure from the normal arrangement, and second, the finding of mirror-imagery adds significant complications to the surgical management of congenital cardiac malformations. The IPCCC had separate codes for heterotaxy and situs inversus; both of these codes are included in the category "Heterotaxy, including isomerism and mirror-imagery".

2. Anomalies of the venous return

This group includes all the anomalies of the venous pole of the heart. Within its 2 subcategories, lesions involving anomalous pulmonary and systemic venous return, the groupings are anatomically coherent.

3. Anomalies of the atria and interatrial communications

This group has an anatomic consistency, concerning the atrial segment of the heart. We excluded, nonetheless, the ostium primum interatrial communications, as well as common atrium, since the phenotypic feature of these entities is the commonality of the atrioventricular junction. We chose to use the term "interatrial communication" (IAC) rather than "atrial septal defect" because the sinus venosus and the coronary sinus defects, although being IAC, are not from an anatomic standpoint defects within the interatrial septum.

4. Anomalies of the atrioventricular junctions and valves

Anatomically, the atrioventricular junctions include the adjacent components of atrial and ventricular musculature and the atrioventricular valves. Within this group, therefore, because of the commonality of the atrioventricular junction, we included the ostium primum defects rather than including these lesions with the other interatrial communications. Because of their wide acceptance, we decided to retain the terms "partial" and "complete" atrioventricular septal defects (AVSD) to distinguish the various forms of common atrioventricular junction, the latter indicating those forms with both atrial and ventricular shunting [13]. These anomalies can all be detected on foetal echocardiography using the four-chamber view.

5. Complex anomalies of the atrioventricular connections

We included the combination of discordant atrioventricular and ventriculo-arterial connections, congenitally corrected transposition of great arteries (TGA) or double discordance, in this group, and not in the group of anomalous ventriculo-arterial connections. This is because the discordant atrioventricular connections represent the more significant feature, clinically, anatomically and surgically, of this complex cardiac anomaly. This group does not, however, include anomalies of the atrioventricular connections in the setting of isomerism or visceral heterotaxy. We also included in this group, although they are not specific entities, criss-cross AV relations and supero-inferior ventricles, because these anomalies result from a rotational malalignment of the atrial and ventricular structures, even if the AV connections can be concordant in these patients [14].

6. Functionally univentricular hearts

Heterogeneous from an anatomic standpoint, all the malformations included in this category have as their common denominator severe hypoplasia of one ventricle. They share the same medical and surgical management, based on one-ventricle repair, and aimed eventually at creation of a total cavopulmonary connection. We chose not to include unbalanced AVSD in this category, on the basis that one of the ventricles could be inappropriately small in these cases but thought to be suitable for biventricular repair [13]. If the patients were considered more suitable for functionally univentricular repair, then it would be more appropriate to categorise the lesions as double inlet ventricle through a common atrioventricular valve. Along the same line, we did not include in this group either those patients with hypoplastic mitral and tricuspid valves, nor those with straddling mitral and tricuspid valves, again considering that, if one ventricle was truly hypoplastic in these settings, the patients would likely be diagnosed as having double inlet ventricle. These lesions can all be detected on fetal echocardiography when using the four-chamber view.

7. Ventricular septal defects (VSD)

This group includes not only isolated VSD's, but also additional (multiple) VSD in the setting of another lesion, including of necessity a VSD, such as tetralogy of Fallot. Anatomically heterogeneous, this group is very important numerically. The anatomic nomenclature of VSD is still controversial. We used in our classification the unified nomenclature system proposed by the Congenital Heart Surgery Nomenclature and Database Project [15]. In addition to the anatomic location of the defect, the IPCCC allows a rough appreciation of the clinical importance of the shunt with the notion of large or small VSD.

8. Anomalies of the ventricular outflow tracts

The anatomic definition of the ventricular outflow tracts is the area of junction between the ventricular and the arterial segments of the heart. This large category includes the anomalies of the subvalvar region, specifically the subpulmonary infundibulum or conus, the left ventricular outflow tract, the arterial valves and their supporting sinuses, and the intrapericardial segments of the great arterial trunks. Within this group we include subcategories for abnormal ventriculo-arterial connections, including TGA, double-outlet right ventricle, double-outlet left ventricle, and concordant ventriculo-arterial connections with parallel rather than spiralling arterial trunks, or anatomically corrected malposition of the great arteries [16,17]. We included tetralogy of Fallot (TOF) with pulmonary atresia rather than stenosis within the subcategory "TOF and variants", rather than in the subcategory of abnormal ventriculo-arterial connections. All other types of atresia of the pulmonary outflow tract, irrespective of the associated lesions, were included into the subcategory "right ventricular outflow tract anomalies", except for pulmonary atresia with intact ventricular septum, which was included in the group "functionally univentricular hearts". Common arterial trunk was classified according to the description of anatomic phenotypes, to avoid the controversies induced by alphanumeric classifications, although this categorization is still a subject of debate and deserves improvement [18].

From the surgical standpoint, usually the two ventricles are of normal size in these lesions, making the defects suitable for biventricular repair.

9. Anomalies of the extrapericardial arterial trunks

This group includes all the anomalies of the great arteries beyond the boundaries of the pericardial cavity. Clinically heterogeneous, their diagnosis is difficult before birth, and tends to be based after birth on CT-scan, MRI, or catheterization.

10. Congenital anomalies of the coronary arteries

These anomalies are rarely isolated, but for clinical reasons pose a major concern when associated with various lesions, particularly abnormalities of the ventricular outflow tracts.

b) The 23 subcategories

We defined 23 subcategories according to the anatomy of the heart and "clinical sense". For example, "Anomalies of atrioventricular junctions and valves" includes 3 subcategories according to the type of atrioventricular valve involved, specifically tricuspid valve, mitral valve, or common atrioventricular valve in the setting of atrioventricular septal defect. We did not include atrioventricular valvar atresia in this subcategory, but placed these patients along with those having functionally univentricular hearts, since their main characteristic is severe hypoplasia of one of the two ventricles, irrespective of the anatomy of atrioventricular junction.

### The coding system

Rather than creating a new code number for each cardiac defect encountered in our population, we chose to attribute one six-digit code of the long list to each item of our list, facilitating in this way the interface with other databases or registries. The choice was sometimes difficult among the numerous codes proposed for one given lesion, and the final decision was made based on principles of simplicity and parsimony, combined with clinical judgement. For example, we chose to code sinus venosus type interatrial communication according to its most frequent form: "sinus venosus defect (interatrial communication) with overriding superior caval vein (superior defect)": 05.05.01.

### Hierarchy used for the coding process

The hierarchy chosen for coding is fundamental, since it places the chosen lesion into *one, and only one*, of our 10 groups. Each case received one single code if the lesion was well-defined, as for example tetralogy of Fallot. On the other hand, multiple codes were used in the following situations:

- Well-defined CHD with associated anomalies (e.g., TOF with left anterior descending coronary artery originating from the right coronary artery). In this case, the aim was to use a hierarchy that would be logical and intuitive (Code n°1 for the main anomaly and Code n°2 for the associated anomaly).

- Complex CHD: Each case could receive up to five codes in order to describe accurately the major elements of the CHD. In this case, we followed the hierarchy commonly used in the clinical process of aggregation in daily practice of paediatric cardiology. For example, complex left heart disease may bring together several anomalies belonging to different main categories in our classification, such as left ventricular hypoplasia, suggesting a functionally univentricular heart, parachute mitral valve, indicating anomalies of the atrioventricular junctions and valves, coarctation of the aorta, an anomaly of the great arteries, a left superior caval vein draining to the coronary sinus, an obvious anomaly of the venous return, and a small perimembranous VSD. The chosen main code would then reflect the planned clinical and surgical management, with the other anomalies then classified randomly. The determination of the major lesion of course, is not always clear-cut, and may be prone to controversy.

The proposed scheme for classification, including the 10 main categories and the 23 subcategories, together with their corresponding IPCCC and ICD-10 codes, is summarized in the Additional File 1. The list of lesions shown in the Additional File 1 is, of course, not exhaustive, but of necessity represents only those encountered among the patients enrolled in the EPICARD study. This list is not fixed, and can easily be expanded using the six-digit codes of the comprehensive long list.

## Results

Table 1 shows the results of applying our suggested classification to the 2867 studied cases from the EPICARD study. Given the level of detail and specificity of the IPCCC codes, four-fifths could be identified with a single code. Of the remaining cases, most could be identified with two codes.

**Table 1:** Number and distribution of IPCCC codes for Congenital Heart Defects (CHD) included in the EPICARD study

Number of codes	Total number of CHD cases*	%
**1**	**2279**	**79.5**
**2**	**405**	**14.1**
**3**	**117**	**4.1**
**4**	**37**	**1.3**
**5**	**27**	**0.9**
**6**	**2**	**0.1**

**All**	**2867**	**100.0**

* including livebirths, stillbirths and pregnancy terminations

In Table 2, we show the distribution of the number of EPICARD cases in our ten selected groups. Ventricular septal defects, with 1,492 instances, accounted for more than half of the cases (52.0%). The second largest group was anomalies of the outflow tracts and arterial valves, with 563 cases, accounting for one-fifth of all lesions (19.6%). The smallest numbers were 9 patients with anomalies of the coronary arteries, 13 with complex anomalies of atrioventricular connections, and 31 with anomalies of venous return, all of these together accounting for less than 2% of the overall series.

**Table 2:** Distribution of the number of CHD cases in the ten categories of the anatomic and clinical classification of congenital heart defects (ACC-CHD) in the EPICARD study

Group	N	%
1. Heterotaxy, including isomerism and mirror-imagery	37	1.3
2. Anomalies of the venous return	31	1.1
3. Anomalies of the atria and interatrial communications	182	6.3
4. Anomalies of the atrioventricular junctions and valves	213	7.4
5. Complex anomalies of atrioventricular connections	13	0.45
6. Functionally univentricular hearts	158	5.5
7. Ventricular septal defects (VSD)	1492	52.0
8. Anomalies of the ventricular outflow tracts (ventriculo-arterial connections)	563	19.6
9. Anomalies of the extrapericardial arterial trunks	169	5.9
10. Congenital anomalies of the coronary arteries	9	0.3

**Total**	**2867**	**100**

In Table 3, we show the number and distribution of IPCCC codes in our chosen groups. For most groups, a single code was sufficient to identify the majority of cases. In particular, approximately 90% of cases classified as anomalies of the atria and interatrial communications, and those falling within anomalies of the venous return, could be identified with a single code. This was also true for more than 70% of cases of anomalies of the outflow tracts and arterial valves and anomalies of the atrioventricular junctions and valves.

**Table 3:** Distribution of the IPCCC codes in the ten categories of the anatomic and clinical classification of congenital heart defects (ACC-CHD) in the EPICARD study

Group	Number of codes	Number of CHD cases	%
1. Heterotaxy, including isomerism and mirror-imagery	1 2 3 4 5 6	1 3 7 10 14 2 **37**	2.7 8.1 18.9 27 37.9 5.4 **100.0**
2. Anomalies of the venous return	1 2 3 4 5 6	28 3 0 0 0 0 **31**	90.3 9.7 0.0 0.0 0.0 0.0 **100.0**
3. Anomalies of the atria and interatrial communications	1 2 3 4 5 6	159 19 4 0 0 0 **182**	87.4 10.4 2.2 0.0 0.0 0.0 **100.0**
4. Anomalies of the atrioventricular junctions and valves	1 2 3 4 5 6	158 44 9 1 1 0 **213**	74.2 20.6 4.2 0.5 0.5 0.0 **100.0**
5. Complex anomalies of atrioventricular connections	1 2 3 4 5 6	5 3 4 0 1 0 **13**	38.5 23.1 30.8 0.0 7.7 0.0 **100.0**
6. Functionally univentricular hearts	1 2 3 4 5 6	100 34 15 3 6 0 **158**	63.3 21.5 9.5 1.9 3.8 0.0 **100.0**
7. Ventricular septal defects (VSD)	1 2 3 4 5 6	1337 135 19 1 0 0 **1492**	89.6 9 1.3 0.1 0.0 0 **100.0**
8. Anomalies of the ventricular outflow tracts (ventriculo-arterial connections)	1 2 3 4 5 6	404 96 43 17 3 0 **563**	71.8 17.1 7.6 3.0 0.5 0.0 **100.0**
9. Anomalies of the extrapericardial arterial trunks	1 2 3 4 5 6	78 68 16 5 2 0 **169**	46.1 40.2 9.5 3 1.2 0.0 **100.0**
10. Congenital anomalies of the coronary arteries	1 2 3 4 5 6	9 0 0 0 0 0 **9**	100.0 0.0 0.0 0.0 0.0 0.0 **100.0**

Those patients classified as having heterotaxy were the only ones almost always requiring more than one IPCCC code for their identification. In addition, 37% of those categorized as having functionally univentricular hearts, and more than half of those coded with anomalies of the extrapericardial arterial trunks and complex anomalies of atrioventricular connections required two or more codes for their full classification.

## Discussion

The classification we are proposing, rather than being new, is no more than a rearrangement of the long list of the existing IPCCC. The rearrangement is based on the cardiac anatomy, along with criteria used for therapeutic management. It is designed to avoid doublets and to reduce ambiguities. It is also intended to facilitate both the coding process and the analysis of the data in the setting of clinical and epidemiological studies.

Despite its worldwide use, the shortcomings of ICD -10 are increasingly recognized. Several studies have shown that the ninth version of ICD, still in use in the United States of America for administrative databases, generates a substantial number of errors and inaccuracies when compared to the IPCCC [3-5]. Although the tenth version of ICD includes 73 individual codes for CHD, versus only 29 in the ninth version, it remains inadequate [2]. For example, the ostium primum defect shares the same code as an atrioventricular septal defect with ventricular and atrial shunting (Q21.2), while IPCCC clearly makes a distinction between the variants of atrioventricular septal defect with common atrioventricular junction. Two other examples, among many, are demonstrative: the code Q25.4 is common to right aortic arch and interrupted aortic arch, while the code Q20.8 is common to right ventricular hypoplasia, left ventricular hypoplasia, and juxtaposition of the atrial appendages. Another important limitation of ICD-10 is the frequent occurrence of the items "other" or "unspecified" noted with the suffix numbers .8 and .9 respectively, this being a source of many inaccuracies.

In contrast, the IPCCC is remarkable by its exhaustiveness and its accuracy, as it was deliberately designed to be inclusive, allowing the different users to choose their preferred term for any specific congenital cardiac malformation [6]. This makes it very detailed, and hence often too complex for use by epidemiologists and clinicians.

In devising our proposed revised classification, our principal motivation was to rearrange the whole spectrum of CHD into a manageable number of categories defined on their anatomy as well as the criteria used for their clinical and surgical management. The classification is designed to use the code numbers in the long list of IPCCC but can accommodate ICD-10 codes.

### Choice of the main categories and subcategories

The categories common to the ACC-CHD and the IPCCC long list are:

- anomalies of venous return or great veins,

- anomalies of the atrioventricular valves and of the atrioventricular junctions

- anomalies of the atria and interatrial communications

- anomalies of the ventricles and of the ventricular septum (except for VSD's).

We deliberately chose to group all types of ventricular hypoplasia within the large category of functionally univentricular hearts, on the basis of their common medical and surgical management. In the IPCCC, left and right ventricular hypoplasia are included in the group of abnormalities of the ventricles, while hypoplastic left heart syndrome and pulmonary atresia with intact ventricular septum, together with double-inlet ventricles, are in the group of anomalous atrioventricular and ventriculo-arterial connections.

We also isolated the anomalies of the coronary arteries as a distinct category, because of their frequency as an associated anomaly and their clinical importance, instead of including them into the anomalies of great vessels, as in the long list of the IPCCC, or into a miscellaneous group as in one version of the short list. We found, nonetheless, very few cases in EPICARD, suggesting either that anomalies of the coronary arteries are frequently missed as part of the final diagnosis of the congenital malformation, or that they are, indeed, very rare.

We created a large category named "anomalies of outflow tracts and arterial valves" in order to merge all the anomalies of the ventricular outflow tracts, including abnormal ventriculo-arterial connections, abnormal arterial valves, and lesions of the intrapericardial arterial trunks. In the IPCCC long and short lists, the abnormal ventriculo-arterial connections, along with the anomalies of atrioventricular connection, are placed in the large group of abnormalities of position and connection of the heart, while the anomalies of the arterial valves and the outflow tracts are included into the group of the anomalies of great vessels and ventriculo-arterial valves. For anatomic, clinical, and surgical reasons, we chose to include these in a single category, which then accounted for about one-fifth of all cases in EPICARD, and at least two-fifths of major cardiac defects when we exclude minor VSD's.

We divided those with heterotaxy, and those with complex anomalies of atrioventricular connections, into two distinct groups, the latter including congenitally corrected transposition or double discordance.

Because of their frequency, we considered patients with ventricular septal defects as a distinct entity. Within this group, we then incorporated the notion of size, and thus of clinical severity, as suggested in the IPCCC. The specific code for a "small" VSD does not exist in the ICD-10.

### The coding process

The hierarchy used for coding complex CHD, although based on the clinical aggregation process reflecting the routine practice of paediatric cardiology, and clear-cut in many cases, might be questionable for some complex associations. In addition, some cardiac anomalies may exist in isolation, as well as in association with various types of CHD. For example, pulmonary atresia was considered as the main cardiac defect in only two situations, those co-existing with tetralogy of Fallot on the one hand, an anomaly of the ventricular outflow tracts, and with an intact ventricular septum on the other hand, included in the group of functionally univentricular hearts, albeit that some of the latter patients can undergo biventricular repair. In other situations, pulmonary atresia is associated with another CHD, with a specific six-digit code. Pulmonary atresia in the setting of congenitally corrected transposition, for example, was coded as double discordance, VSD, and associated pulmonary atresia.

We often chose to code the associated anomalies as second, third, and fourth added codes, rather than using the different code numbers listed in the IPCCC, simply because they cannot be totally inclusive. There are, nonetheless, some exceptions to this rule, with AVSD with TOF, for example, having a specific code number.

## Conclusion

The long list of IPCCC is sufficiently detailed and precise to code satisfactorily the entire spectrum of congenital cardiac disease. It is, however, complex, including literally thousands of codes, which may hinder its use in clinical and epidemiological studies. Our suggested re-classification separates the entire spectrum of CHD into a manageable number of categories that use the code numbers of the IPCCC long list, and are based on anatomic criteria, as well as those used for clinical and surgical management. It can be useful for clinical and epidemiologic studies that aim to evaluate the outcomes of patients with CHD, and those seeking to assess the role of prognostic factors. It can also be helpful in epidemiologic studies of risk factors for CHD, and in particular those aimed at exploring specific associations that may exist between risk factors and different types of CHD. Indeed, it was found to be useful in a population-based study about the risk of CHD associated with assisted reproductive technologies [19]. The proposed classification can also provide a structure for various clinical and epidemiologic databases.

## Abbreviations

ACC-CHD: anatomic and clinical classification of congenital heart defects; AVSD: atrioventricular septal defect; CHD: congenital heart defects; CT-scan: computed tomography: IAC: interatrial communication; ICD-10: International classification of Diseases, 10^th ^version; IPCCC: international paediatric and congenital cardiac code; MRI: magnetic resonance imaging; RACHS-1: Risk Adjustment in Congenital Heart Surgery; STS/EACTS: Society of Thoracic Surgeons/European Association for Cardio-thoracic Surgery; TGA: transposition of great arteries; TOF: tetralogy of Fallot; VSD: ventricular septal defect.

## Competing interests

The authors declare that they have no competing interests.

## Authors' contributions

LH conceived the classification and drafted the manuscript. BK and FG conceived and organized the EPICARD study, participated in the interpretation of data, helped to draft, and critically revised the manuscript. RHA made substantial contributions when critically revising the manuscript for important intellectual content. BK supervised, and NL and ACT conducted the statistical analysis of the data and interpreted the results. DB participated in designing the classification and drafting the manuscript, and critically revised the manuscript for important intellectual content. All authors read and approved the final manuscript.

## Supplementary Material

Additional file 1 **This table displays our anatomic and clinical classification of congenital heart defects (ACC-CHD) with the corresponding IPCCC and ICD-10 codes**. The list of lesions shown in the Additional File is, of course, not exhaustive, but of necessity represents only those encountered among the patients enrolled in the EPICARD study. This list is not fixed, and can easily be expanded using the six-digit codes of the comprehensive long list. Click here for file
