# Supplementary material for: Population-based evaluation of a suggested anatomic and clinical classification of congenital heart defects based on the International Paediatric and Congenital Cardiac Code
Source: Orphanet J Rare Dis. 2011 Oct 3;6:64. doi: 10.1186/1750-1172-6-64 (PMC3198675; doi:10.1186/1750-1172-6-64)
Supplement: Additional file 1 — This table displays our anatomic and clinical classification of congenital heart defects (ACC-CHD) with the corresponding IPCCC and ICD-10 codes. The list of lesions shown in the Additional File is, of course, not exhaustive, but of necessity represents only those encountered among the patients enrolled in the EPICARD study. This list is not fixed, and can easily be expanded using the six-digit codes of the comprehensive long list. [file 1750-1172-6-64-S1.DOC]

**Additional File**. Anatomic and clinical classification of congenital heart defects (ACC-CHD) with the corresponding IPCCC and ICD-10 codes.

| CONGENITAL HEART DEFECTS | IPCCC | ICD-10 |
| --- | --- | --- |
| 1. Heterotaxy, including isomerism and mirror-imagery  - Heterotaxy syndromes (heterotaxia syndrome, visceral heterotaxy: abnormal arrangement of thoraco-abdominal organs)  - Isomerism of atrial appendages  - Left  - Right  - Situs inversus (mirror-imaged arrangement) | 03.01.02  01.03.08  01.03.03  01.03.02  03.01.03 | Q89.3  Q20.6  Q20.6  Q20.6  Q89.3 |
| 2. Anomalies of the venous return  2.1. Anomalies of the systemic venous return (congenital)  - Congenital anomaly of the superior vena cava (SVC)  - Absent right SVC  - Retro-aortic innominate vein  - Left SVC persisting to coronary sinus  - Left SVC persisting to left-sided atrium  - Congenital anomaly of the coronary sinus  - Partially unroofed coronary sinus  - Totally unroofed coronary sinus  - Coronary sinus orifice atretic  - Congenital anomaly of the inferior vena cava (IVC)  - Azygos continuation of the IVC  - Right-sided azygos continuation of the IVC  - Left-sided azygos continuation of the IVC  - Congenital anomaly of the hepatic veins  - Separate hepatic and IVC venous return  2.2. Anomalies of the pulmonary venous connections  - Anomalous pulmonary venous connection  - Totally  - Supracardiac  - Intracardiac  - Infracardiac  - Mixed  - Partially  - Congenital pulmonary vein stenosis  - Congenital pulmonary vein hypoplasia  - Pulmonary vein(s) atresia  - Scimitar syndrome | 04.01.09  04.01.05  04.00.04  04.01.01  04.01.02  04.04.05  04.04.01  04.04.02  04.04.03  04.03.08  04.03.10  04.03.02  04.03.01  04.02.04  04.02.06  04.08.07  04.08.05  04.06.00  04.08.10  04.08.20  04.08.30  04.07.01  04.08.01  04.08.08  04.08.02  01.01.16 | Q26.9  Q26.8  Q26.9  Q26.1  Q26.1  Q21.1  Q21.1  Q21.1  Q21.1  Q26.9  Q26.8  Q26.8  Q26.8  Q26.8  Q26.8  Q26.4  Q26.2  Q26.2  Q26.2  Q26.2  Q26.2  Q26.3  Q26.8  Q26.8  Q26.8  Q26.8 |
| 3. Anomalies of the atria and interatrial communications (IAC)  3.1. Cor triatriatum  - Cor triatriatum dexter (obstructive Eustachian valve)  3.2. IAC  - patent oval foramen  - ostium secundum type  - sinus venosus type (superior)  - coronary sinus type  - for ostium primum type go to section 4.3  3.3. Juxtaposition of the atrial appendages (JAA)  - Left JAA  - Right JAA | 05.02.01  05.01.21  05.04.01  05.03.01  05.04.02  05.05.01  05.05.03  05.00.01  05.01.06  05.02.04 | Q24.2  Q24.2  Q21.1  Q21.1  Q21.1  Q21.1  Q21.1  Q20.8  Q20.8  Q20.8 |
| 4. Anomalies of the atrioventricular junctions and valves  4.1. Congenital anomalies of the tricuspid valve (TV)  - Ebstein’s malformation  - Associated with discordant AV connections  - TV agenesis (unguarded tricuspid orifice)  - Dysplastic tricuspid valve  - Congenital tricuspid insufficiency  - Cleft of the TV  - Congenital valvar tricuspid stenosis  - Tricuspid annular hypoplasia  - Straddling TV  - Overriding TV  - Anomaly of the TV subvalvar apparatus 4.2. Congenital anomalies of the mitral valve (MV)  - Dysplastic MV  - Congenital mitral insufficiency  - Isolated cleft of the MV (not AVSD type)  - Congenital MV prolapse  - Congenital mitral valvar stenosis  - Supravalvar mitral ring  - Mitral annular hypoplasia  - Parachute MV  - Double mitral orifice  - Straddling MV  - Overriding MV  - Congenital anomaly of the MV subvalvar apparatus  - Accessory mitral tissue  4.3. Atrioventricular septal defects (AVSD)  - “Complete” AVSD (ventricular and atrial components with common AV orifice)  - with left ventricular (LV) hypoplasia  - with right ventricular (RV) hypoplasia  - with Tetralogy of Fallot  - “Partial” AVSD  - ostium primum type (atrial shunting only)  - “intermediate” or “transitional” type (atrial shunting and restrictive ventricular shunting)  - cleft of the left atrioventricular (AV) valve in AVSD (“mitral cleft” in AVSD)  - common atrium (virtual absence of atrial septum)  - isolated ventricular component (ventricular shunting only) | 06.01.34  06.01.75  06.01.32  06.01.39  06.01.25  06.01.36  06.01.07  06.01.04  06.01.09  06.01.05  06.01.12  06.02.40  06.02.25  06.02.36  06.02.72  06.02.07  05.02.02  06.02.04  06.02.56  06.02.33  06.02.09  06.02.05  06.02.21  06.02.39  06.06.09  06.07.05  06.07.06  01.01.20  06.06.01  06.06.10  06.05.69  05.06.01  06.06.08 | Q22.5  Q22.5+Q20.5  Q22.9  Q22.8  Q22.8  Q22.8  Q22.4  Q22.4  Q22.8  Q22.8  Q22.8  Q23.8  Q23.3  Q23.9  I34.1  Q23.2  Q20.8  Q23.2  Q23.8  Q23.9  Q23.8  Q23.8  Q23.8  Q23.9  Q21.2  Q21.2+Q20.8  Q21.2+Q20.8  Q21.2+Q21.3  Q21.2  Q21.2  Q23.9  Q21.2  Q21.2 |
| 5. Complex anomalies of atrioventricular connections  - Congenitally corrected transposition of the great arteries (double discordance)  - Criss-cross atrioventricular connections  - Supero-inferior ventricles | 01.01.03  02.03.03  02.04.00 | Q20.5  Q24.8  Q24.8 |
| 6. Functionally univentricular hearts  6.1. Double-inlet ventricle (DIV)  - with 2 atrioventricular valves  - Double-inlet right ventricle  - Double-inlet left ventricle  - Right-sided AV valve in DIV atretic (imperforate)  - Left-sided AV valve in DIV atretic (imperforate)  - Common AV orifice in double-inlet ventricle  6.2. Absence of one atrioventricular connection  - Absent left-sided AV connection (mitral atresia)  - Absent right-sided AV connection (tricuspid atresia)  6.3. Left ventricular (LV) hypoplasia  - Hypoplastic left heart syndrome  - Mitral valve atretic (imperforate)  - Ventricular imbalance with dominant RV and hypoplastic LV  6.4. Right ventricular (RV) hypoplasia  - Pulmonary atresia with intact ventricular septum  - Tricuspid valve atretic (imperforate): congenital  - Hypoplastic right heart syndrome  - Ventricular imbalance with dominant LV and hypoplastic RV  - Uhl’s anomaly | 01.01.14  01.06.01  01.04.03  01.04.04  06.03.02  06.04.02  01.06.02  01.04.20  01.04.19  07.07.00  01.01.09  06.02.02  07.08.42  07.02.00  01.01.07  06.01.02  07.02.10  07.08.41  07.01.06 | Q20.4  Q20.4  Q20.4  Q20.4  Q20.4  Q20.4  Q20.4  Q23.2  Q22.4  Q20.8  Q23.4  Q23.2  Q20.8  Q20.8  Q22.0  Q22.4  Q22.6  Q20.8  Q24.8 |
| 7. Ventricular septal defects (VSD)  - Perimembranous VSD  - Perimembranous VSD, small  - Perimembranous VSD with posterior (inlet) extension  - Malalignment (infundibular, conoventricular) VSD (with malaligned outlet (conal) septum)  - anterior malalignment VSD (Fallot type)  - posterior malalignment VSD (aortic arch obstruction type)  - Doubly committed (subarterial, conal) VSD  - Muscular VSD  - Muscular VSD, small  - Inlet VSD, not associated with a common AV junction  - Multiple VSDs  - Associated with aortic insufficiency (prolapsed aortic leaflet) | 07.10.00  07.10.01  07.15.03  07.10.02  07.10.12  07.10.08  07.10.06  07.12.01  07.11.01  07.15.02  07.14.06  07.15.04  07.14.07 | Q21.0  Q21.0  Q21.0  Q21.0  Q21.0  Q21.0  Q21.0  Q21.0  Q21.0  Q21.0  Q21.0  Q21.0  Q21.0 |
| 8. Anomalies of the ventricular outflow tracts (ventriculo-arterial connections)  8.1. Transposition of the great arteries (TGA)  - TGA with intact ventricular septum  - TGA (discordant ventriculo-arterial connections)  (for complex TGA add any associated lesion to this code)  8.2. Other abnormal ventriculo-arterial (VA) connections  - Double outlet right ventricle (DORV)  - with subaortic VSD  - with subpulmonary VSD  - with non-committed VSD  - with doubly committed VSD  - with intact ventricular septum  - Fallot type (subaortic or doubly committed VSD + subpulmonary stenosis)  - Double outlet left ventricle  - Concordant VA connections with parallel great arteries (Anatomically corrected malposition of the great arteries)  8.3. Tetralogy of Fallot and variants  - Tetralogy of Fallot (TOF)  - Tetralogy of Fallot with pulmonary atresia  - with MAPCAs (collaterals)  - Absent pulmonary valve syndrome  - with non-Fallot VSD  - with Fallot-type VSD  8.4 Anomalies of the intrapericardial arterial trunks  - Common arterial trunk (truncus arteriosus)  - with common origin of PAs (type I)  - with separate origin of PAs (type II)  - with isolated (discontinuous) PAs  - with aortic arch obstruction  - Aorto-pulmonary window  - Pulmonary artery ( PA) from ascending aorta  - Right PA from ascending aorta  - Left PA from ascending aorta  - Anomalies of the ascending aorta  - Atresia of the ascending aorta  - supravalvar aortic stenosis  - Aortic sinus of Valsalva aneurysm  - aorto-left ventricular tunnel  - Supravalvar pulmonary stenosis  8.5. Left ventricular outflow tract (LVOT) and aortic valvar anomalies  - valvar aortic stenosis: congenital  - bicuspid aortic valve  - dysplastic aortic valve  - aortic valve atresia  - LVOT obstruction  - subvalvar aortic stenosis (fibromuscular tunnel)  - subaortic fibromuscular shelf  - congenital aortic insufficiency  8.6. Right ventricular outflow tract (RVOT) and pulmonary valvar anomalies  - pulmonary valvar stenosis: congenital  - dysplastic pulmonary valve  - bicuspid pulmonary valve  - pulmonary atresia associated with other CHD  (add additional code for any associated lesions(s))  - subpulmonary stenosis  - RVOT obstruction (subpulmonary)  - stenosis of mouth of the infundibulum  - RVOT obstruction due to malaligned outlet septum  - double chambered right ventricle | 01.01.02  01.05.01  01.01.04  01.01.41  01.01.18  01.01.19  01.01.23  01.01.24  01.01.17  01.05.03  01.05.10  01.01.01  01.01.26  01.01.25  09.05.28  09.05.25  09.01.01  09.01.02  09.01.03  09.01.11  09.01.12  09.04.01  09.09.08  09.09.03  09.09.05  09.16.03  09.16.00  09.18.01  09.17.02  09.07.13  09.15.01  09.15.22  09.15.09  09.15.06  07.09.01  07.09.16  07.09.03  09.15.07  09.05.04  09.05.24  09.05.32  09.05.11  07.05.30  07.05.12  07.05.02  07.05.04  07.03.01 | Q20.3  Q20.3  Q20.1  Q20.1  Q20.1  Q20.1  Q20.1  Q20.1  Q20.1  Q20.2  Q25.9  Q21.3  Q22.0+Q21.0  Q22.0+Q21.0  Q22.3  Q22.3  Q20.0  Q20.0  Q20.0  Q20.0  Q20.0  Q21.4  Q25.7  Q25.7  Q25.7  Q25.2  Q25.3  Q25.4  Q25.4  Q25.6  Q23.0  Q23.1  Q23.8  Q23.0  Q24.4  Q24.4  Q24.4  Q23.1  Q22.1  Q22.3  Q22.3  Q22.0  Q24.3  Q24.3  Q24.3  Q24.3  Q24.8 |
| 9. Anomalies of the extrapericardial arterial trunks  9.1. Patent ductus arteriosus  - patent ductus arteriosus, atypical (abnormal origin)  9.2. Coarctation of the aorta  - coarctation of the aorta, abdominal  - Hypoplasia of the aortic arch (tubular)  9.3 Interruption of the aortic arch  - type A: distal to subclavian artery  - type B: between subclavian and common carotid  - type C: between carotid arteries  9.4. Anomalies of the aortic arches  - right aortic arch  - cervical aortic arch  - aberrant origin subclavian artery  - aberrant origin of right subclavian artery  - aberrant origin of left subclavian artery  - arterial duct from left innominate artery  - double aortic arch  - persistent fifth aortic arch  9.5. Anomalies of the pulmonary artery (PA) and its branches  - Pulmonary arterial stenosis  - Right PA stenosis  - Left PA stenosis  - Pulmonary arterial hypoplasia  - Right PA hypoplasia  - Left PA hypoplasia  - Pulmonary artery absent  - Right PA absent  - Left PA absent  - Pulmonary artery from patent arterial duct  - Right PA from patent arterial duct  - Left PA from patent arterial duct  - Pulmonary arterial sling | 09.27.21  09.27.41  09.29.01  09.29.06  09.29.11  09.29.31  09.29.32  09.29.33  09.29.34  09.28.15  09.28.06  09.30.01  09.30.02  09.30.04  09.27.47  09.28.09  09.28.08  09.10.01  09.10.25  09.10.26  09.10.11  09.10.12  09.10.13  09.10.21  09.10.22  09.10.23  09.09.11  09.09.02  09.09.04  09.09.06 | Q25.0  Q25.8  Q25.1  Q25.1  Q25.4  Q25.4  Q25.4  Q25.4  Q25.4  Q25.4  Q25.4  Q27.8  Q27.8  Q27.8  Q25.8  Q25.4  Q25.4  Q25.6  Q25.6  Q25.6  Q25.7  Q25.7  Q25.7  Q25.7  Q25.7  Q25.7  Q25.7  Q25.7  Q25.7  Q25.7 |
| 10. Congenital anomalies of the coronary arteries  - Anomalous origin of left coronary artery from pulmonary artery (ALCAPA)  - Single coronary artery supplying all the heart  - Coronary orifice stenosis: congenital  - Coronary orifice atresia  - Aberrant course of left anterior descending from right coronary artery across RVOT  - Right ventricular myocardial sinusoids  - Coronary artery fistula: congenital  - coronary fistula to pulmonary artery | 09.46.00  09.41.03  09.43.13  09.44.05  09.44.06  09.43.04  07.01.13  09.45.16  09.45.06 | Q24.5  Q24.5  Q24.5  Q24.5  Q24.5  Q24.5  Q24.8  Q24.5  Q24.5 |
